# Profiling of *Bacillus cereus* on Canadian grain

**DOI:** 10.1371/journal.pone.0259209

**Published:** 2021-11-04

**Authors:** Niradha Withana Gamage, Janice Bamforth, Tehreem Ashfaq, Kathryn Bernard, Tom Gräfenhan, Sean Walkowiak

**Affiliations:** 1 Canadian Grain Commission, Government of Canada, Winnipeg, Canada; 2 Public Health Agency of Canada, National Microbiology Laboratory, Government of Canada, Winnipeg, Canada; Northumbria University, UNITED KINGDOM

## Abstract

Microorganisms that cause foodborne illnesses challenge the food industry; however, environmental studies of these microorganisms on raw grain, prior to food processing, are uncommon. *Bacillus cereus sensu lato* is a diverse group of bacteria that is common in our everyday environment and occupy a wide array of niches. While some of these bacteria are beneficial to agriculture due to their entomopathogenic properties, others can cause foodborne illness; therefore, characterization of these bacteria is important from both agricultural and food safety standpoints. We performed a survey of wheat and flax grain samples in 2018 (n = 508) and 2017 (n = 636) and discovered that *B*. *cereus* was present in the majority of grain samples, as 56.3% and 85.2%, in two years respectively. Whole genome sequencing and comparative genomics of 109 presumptive *B*. *cereus* isolates indicates that most of the isolates were closely related and formed two genetically distinct groups. Comparisons to the available genomes of reference strains suggested that the members of these two groups are not closely related to strains previously reported to cause foodborne illness. From the same data set, another, genetically more diverse group of *B*. *cereus* was inferred, which had varying levels of similarity to previously reported strains that caused disease. Genomic analysis and PCR amplification of genes linked to toxin production indicated that most of the isolates carry the genes *nheA* and *hbID*, while other toxin genes and gene clusters, such as *ces*, were infrequent. This report of *B*. *cereus* on grain from Canada is the first of its kind and demonstrates the value of surveillance of bacteria naturally associated with raw agricultural commodities such as cereal grain and oilseeds.

## Introduction

*Bacillus cereus sensu lato* (*B*. *cereus*) is a group of Gram-positive, rod-shaped, spore forming bacteria, which occupy diverse lifestyles and ecological niches, which is facilitate by its diversity at the gene and genome level [1]. These bacteria and their propagules are present in soil, dust, plants, water, feces, and animal guts [2–4]. In agricultural, strains of these bacteria can associate with the rhizosphere to promote plant growth and are used as biocontrols to manage insect pests and microbial diseases in wheat fields [1]. Other strains of the bacteria are becoming an area of concern in the food industry because they are able to cause foodborne illness and their hardiness makes them difficult to suppress [5–11]. The diversity and ubiquity of *B*. *cereus* also makes strain monitoring challenging. *Bacillus cereus* is the third leading causes of foodborne illnesses in Europe and causes an estimated 63,400 foodborne illnesses per year in the United States [12, 13]. As the human population continues to rise and our food production and processing intensify, it will be of utmost importance to understand the various sources of these bacteria, as well as their diversity.

Despite their close phylogenetic relationship, *B*. *cereus* is comprised of at least eight distinct species with a broad pathogenicity spectrum [14]. One of the most studied species of this group is *B*. *anthracis*, the causative agent of anthrax [15]. Other common species include *B*. *cereus sensu stricto*, an opportunistic pathogen capable of causing food poisoning and other ailments [16], and *B*. *thuringiensi*s, an entomopathogen widely used as biological control agent, specifically against insects for its production of *Bt* toxin [17]. There are six additional species that can be distinguished by rhizoidal growth patterns (*B*. *mycoides* and *B*. *pseudomycoides*) [18], cytotoxicity and thermotolerance (*B*. *cytotoxicus*) [19], ability to cause food spoilage (*B*. *weihenstephanensis*) [20], utility as a probiotic in animal nutrition (*B*. *toyonensis*) [18]. Recently, *B*. *gaemokensis* [21], *B*. *manliponensis* [22], *B*. *bingmayongensis* [23] and *B*. *wiedmannii* [24] have also been suggested as distinct species within this group. Together, *B*. *cereus* is a large group of bacteria that includes several species with unique ecological niches and disease potentials.

From a foodborne illness perspective, *B*. *cereus* can cause diarrheal and an emetic syndrome. These diseases are generally mild and self-limiting; however, more severe infections require hospitalization, and in rare cases *B*. *cereus* can cause other diseases/infections or become fatal [7, 25, 26]. Four *B*. *cereus* exotoxins have been clearly associated with these diseases. Diarrhea has been associated with heat-labile enterotoxins, including hemolytic enterotoxin hemolysin BL (Hbl), non-hemolytic enterotoxin (Nhe), and cytotoxin K (Cytk) [27–29]. These toxins are thought to be produced after the outgrowth of spores, are taken up with contaminated foods, and become active in the small intestine. The emetic syndrome is due to a single depsipeptide toxin, cereulide, which is encoded by the *ces* gene cluster [30]. The protein is stable at pH 2–11 and is resistant to high temperatures [30, 31]. Therefore, it may not be affected by the passage through the gastrointestinal tract, by food processing, or during heat treatment of contaminated food samples. Intoxication related to cereulide can be life threatening and has been linked to severe clinical manifestation, such as acute liver failures and acute encephalopathy [26].

Not all strains or species of *B*. *cereus* can produce these toxins and their identification is important for determining their disease potential. The most commonly used method in routine diagnostics of *B*. *cereus* is the detection and quantification of colonies on selective culture media according to international standards [32]. However, this traditional diagnostic method does not allow for proper assignment of the species within *B*. *cereus* group or for the identification of toxins that they may produce. Therefore, DNA-based identification methods are gaining increasing importance in routine diagnostics [33–36].

The availability of modern DNA-based testing methods, including next-generation sequencing, allow for the detailed characterization of microbes at any stage of the food supply chain; however, few studies have explored microbes in raw grain commodities. Grain crops are typically grown in the fields, where they are exposed to soil, which can be considered as the initial contamination source for spore forming organisms such as *B*. *cereus* [1, 37]. Once harvested, the grain can then be exposed to various contamination sources during storage and handling before it reaches milling and food processing facilities. Therefore, it is possible that *B*. *cereus* cells or spores may accompany the grain before it gets processed. *B*. *cereus* can survive for extended period of time in low moisture environments, which makes the bacteria particularly well-suited for contaminating dry grains. *Bacillus cereus* spores and vegetative forms can be inhabitants of many plants, including cereals and their derivatives [1, 6]. The objective of the present study was to perform genomic characterization of *B*. *cereus* from wheat and flax grains that are produced annually for human and animal consumption.

## Materials and methods

### Enrichment and isolation of *B*. *cereus* from raw grains

In 2017 and 2018, wheat and flax grain samples were received from across Canada. Samples were either obtained from the Harvest Sample Program (HSP) of the Canadian Grain commission or were from cargos that had gone through the grain handling system (i.e. processed at grain elevators and transported by truck and/or rail) and are being prepared for export. Wheat samples from HSP belonged to two wheat classes that are defined according to the Canadian grain registration system, Canadian Western Red Spring (CWRS) and Canadian Eastern Red Spring (CERS) and originated from farms across Canada. Canadian Western Red Spring (CWRS) wheat is the most widely grown wheat in Canada and is produced mostly in the prairies (Manitoba, Saskatchewan, and Alberta), while Canadian Eastern Red Spring (CERS) is from Eastern Canada (Atlantic Canada, Quebec, and Ontario). Cargo samples include CWRS grain samples and accompanying dockage samples (any material intermixed with grain, other than kernels of grain), which we analyzed seperately. Upon receipt and prior to any other handling or processing, grain samples were sub-sampled into sample bags, which were then stored at -30°C. Grain sub-samples were processed for bacterial culturing and enrichment using a modified protocol revised from Health Canada’s Compendium of Analytical Methods for the Microbiological Analysis of Foods (MFLP-52, Nov 2014), which is optimized for the enrichment of diverse bacteria from raw commodities.

Briefly, to enrich for bacteria in the samples, 50 g of grain was transferred into sterile blender bags with filters (Innovation Diagnostics) and 125 mL buffered peptone water was added to each sample. Samples were then incubated for 24 h at 37°C. Next, 125 mL of 10% of tryptone soya broth (TSB) was added to the sample bags and samples were homogenized using a pulsifier (Microgen Bioproduct). After the samples were pulsified, an additional 200 mL of 10% TSB was added to each sample, and samples were incubated at 42°C for 4 h. After incubation, 5 mL of vancomycin (10 μg/mL) and cefsulodin (3 μg/mL) were added to each of the samples, which were then incubated at 42°C for 16 h. Sample bags were then sealed and thoroughly mixed. The liquid portion of the enriched samples were then aliquoted for bacterial testing.

Enriched samples from 2017 were plated onto Petri-dishes containing Chromagar (Alere Inc, CA) and incubated at 42°C for approximately 16 h. On Chromagar, *B*. *cereus* colonies are expected to appear blue with a white halo, allowing them to be differentiated from other bacteria that may be present. Colonies were then preserved in 15% glycerol at -140°C. At a later date, 10μl of the frozen culture was re-plated on new plate of Chromagar and confirmed the colony morphology and purity again prior to DNA extraction.

Samples from 2018 were tested for *B*. *cereus* directly by real-time PCR. DNA was extracted from the enriched cultures using the Blood and Tissue kit (Qiagen), which was automated using a QIAcube (Qiagen). DNA concentrations were determined using the Qubit dsDNA (double-stranded DNA) Broad Range (BR) assay kit (Invitrogen) and DNA quality was measured spectrophotmetrically by Nanodrop 2000 (Thermofisher Scientific). Samples were tested for the presence of *B*. *cereus* by real-time PCR using the Ba primer set (S1 Table) as in [33], with slight modifications. Each 20 μL reaction contained 10 μL SsoAdvanced Universal SYBR green supermix (BioRad), 2 μL of the template DNA, and target specific primers (S1 Table). Amplification was carried out on a CFX96 Optics module (BioRad) and data were analyzed using CFX Manager software (Bio-Rad).

### DNA isolation and PCR testing for toxin genes

Further DNA testing and genome sequencing was performed on 109 strains of *B*. *cereus* isolated from the grain samples (S2 Table). Ninety-nine (99) strains of *B*. *cereus* originated from Canada Western Red Spring (CWRS) wheat (*Triticum aestivum*), two (2) strains were from Canada Eastern Red Spring (CERS) wheat, three (3) of them were from wheat dockage, and three (3) were isolated from flax (*Linum usitatissimum*). Further, two (2) of the previously lab confirmed *B*. *cereus* strains (00–0028 and 05–0322) from the National Microbiology Laboratory NML, Winnipeg were also included in the analysis.

Prior to DNA isolation, *B*. *cereus* strains were grown and sub-cultured on *B*. *cereus* Chromagar plates as described above. To prepare colonies for DNA isolation, 3 mL 0.9% NaCl solution was applied onto the surface of each agar plate and the resulting suspension was aspirated with a pipette and transferred into a reaction tube. Genomic DNA (gDNA) was purified from the isolates using Qiagen Blood and Tissue kit (Qiagen), which was automated using the QIAcube (Qiagen), according to the protocol recommended by the manufacturer for the Gram positive bacteria. All bacterial samples (109) were screened for the presence of five virulence genes (*hbID*, *nheA*, *ces*, *cytk1* and *cytk2*) using real-time PCR as in [34–36] (S1 Table), with slight modifications, as described for the Ba primer set.

### Whole genome sequencing, assembly, and annotation

To capture the genomic diversity of the suspected *B*. *cereus* isolates, we performed whole genome sequencing (S2 Table). Genomic DNA was used to construct NEBNext Ultra II libraries for 94 CWRS samples obtained from harvest year 2018 according to the manufacturer’s instructions. These samples were sequenced using a single lane of an Illumina HiSeqX instrument, which generated 150 bp paired-end reads. Paired-end sequencing reads were then trimmed using the Trimmomatic [38] with options to remove Illumina adapter sequences and to have an average quality score of 30. *De novo* genome assemblies were then generated for each strain using shovill (https://github.com/tseemann/shovill). Two (2) of the suspected cereulide positive samples (BC35N and BC88N) from 2018 were particularly interesting due to their toxin profiles and were suspected to contain the *ces* toxin genes. These two samples and were instead sequenced using Oxford Nanopore Technology R9 flow-cells, and their genomes assembled using canu [39]. Genomes of the rest of the 13 samples from harvest year 2017 and from the National Microbiology Laboratory (NML), Canada, were sequenced on a PacBio RS II sequencer and their genomes assembled at Genome Quebec, Canada. Assembly statistics were generated for each of the genomes using QUAST [40], and gene predictions and annotations were performed using PROKKA [41]. Multilocus sequence typing (MLST) was then performed using PubMLST database and default paramaters of mlst v2.19.0.

### Whole genome comparisons

The genomes were compared to bacterial genome databases using Refseq_masher matches (https://github.com/phac-nml/refseq_masher). The top five matches by Refseq_masher were then downloaded from RefSeq database at the National Center for Biotechnology Information (NCBI) and aligned to each of the genomes using ‘nucmer’ from the software MUMmer [42], with minimum match -l set to 250. The percent genome alignment to each of the references were then extracted using ‘dnadiff’ and representative reference genomes were used for clustering analysis in R using the ‘ComplexHeatmap’ package. Nucleotide variants were identified from the ‘nucmer’ alignments using the genome of the emetic *B*. *cereus* strain AH187 (Accession: GCF_000021225.1) as a reference. Variants were filtered to remove variants with a minor allele frequency <0.05. BC14 and BC89 were also removed from the analysis because they were off-target species that had poor alignment and variant calling to the other genomes. Variants were then subject to principal component and 1,000 iterations of hierarchical clustering analysis in R using ‘prcomp’ and ‘pvclust’, respectively. Based on the scree plot of the nucleotide variant data, seven components were selected for clustering analysis (S1 Fig).

### Analysis of toxin genes, plasmids and antimicrobial genes

To examine the presence/absence of gene homologues with implications in food-borne illness and insecticidal activity (S3 Table), sequences were queried against the whole genome sequences from this study using tBLASTn. BLAST hits were filtered to have >70% alignment length, >50% amino acid sequence identity and <10^−5^ e-value and binary matrix was used to assign 1 for the presence and 0 for the absence of the gene. Since cytK1 and cytK2 are similar in sequence, BLASTn was performed using the primer sequences that distinguish the two toxins (S1 Table). In parallel, we mapped reads directly to the genes of interest using Kma v1.3.23 (https://bitbucket.org/genomicepidemiology/kma/src/master/) using the default parameters recommended for each sequencing technology; genes were considered present if identified with a percent identity and coverage of 90% or greater. To detect plasmids and antimicrobial resistance genes, the staramr tool (https://github.com/phac-nml/staramr) was used to scan bacterial genome contigs against the ResFinder, PointFinder, and PlasmidFinder databases.

## Results and discussion

### *B*. *cereus* is prevalent on wheat grain

The incidence of *B*. *cereus* in grain was assessed by screening flax grains, wheat grains, and dockage from wheat grain samples. The Chromagar culture-based method was implemented in 2017, whereas real-time PCR was used in 2018 (Table 1).

**Table 1:** *B*. *cereus* suspected samples detected by culturing in Chromagar (2017) and by real-time PCR (2018).

Year and Detection Method	Sample Type	Total Samples Processed	*B*. *cereus* Positives	% Positives
2018 real-time PCR	CWRS HSP	231	173	74.89%
	CERS HSP	37	25	67.57%
	CWRS Cargo Grain	124	39	31.45%
	CWRS Cargo Dockage	116	49	42.24%
	**Total 2018**	**508**	**286**	**56.29%**
2017 Chromagar	CWRS HSP	282	265	93.97%
	CERS HSP	111	107	96.40%
	CWRS Cargo Grain	81	43	53.09%
	CWRS Cargo Dockage	65	22	33.84%
	Flax HSP	97	96	98.96%
	**Total 2017**	**636**	**542**	**85.22%**

Samples included Canada Western Red Spring (CWRS) and Canada Eastern Red Spring (CERS) wheat, dockage (any material intermixed with grain, other than kernels of grain), and flax. The samples were obtained from the Harvest Sample Program (HSP) or from cargos.

Of the 636 samples tested in 2017, 85.22% tested positive for *B*. *cereus* using the Chromagar method (Table 1). The flax samples from 2017 had the highest level of suspected *B*. *cereus* samples; however, the positive rate in Canadian Western Red Spring (CWRS) and Canadian Eastern Red Spring (CERS) samples also had high incidence. Real-time PCR of 508 bacterial samples from 2018 identified fewer positive samples, 56.29% than in 2017 (Table 1). In 2018, the highest incidence of *B*.*cereus* positive samples were detected in CWRS HSP and CERS HSP respectively. Nevertheless, we observed the same trend of reduced *B*. *cereus* positive samples in the cargo samples, when compared to HSP samples in both years (Table 1).

It is unclear if the differences observed between 2017 and 2018 are the due to sensitivity and accuracy differences between real-time PCR and Chromagar detection methods or the result of variation in bacterial levels in the grain from the two years. Nevertheless, our findings indicate that incidence of *B*. *cereus* on wheat and flax grains is high, which is expected given the ubiquity of these bacteria in our environment [2]. Our results are consistent with reports from other studies that detected *B*. *cereus* in 81% of incoming wheat samples to be used for milling [11]. Is important to note that the results from this study only reflect incidence (presence/absence) and therefore do not reflect the amount of *B*. *cereus* in the sample, since culturing enriched the bacteria present. Future studies of raw samples without culturing/enrichment would be required to provide a true count of the bacteria in the original grain samples. Other studies indicate that, while present, the levels of *B*. *cereus* in grain are low, and the number of samples testing positive is reduced after grain conditioning [11]. Our observation of reduced incidence of *B*. *cereus* in cargo samples is interesting and suggests that levels of the bacteria may be decreasing as grain continues through the supply chain. While *B*. *cereus* has been reported in cereals previously [6, 11], it is possible that bacterial levels may be higher on the grain immediately after harvest due to sources of bacteria from the field, and that these levels decrease over time as a result of safe storage, handling, transport, and cleaning of the grain.

### Grain harbors diverse strains of *B*. *cereus*

To better capture the diversity of the suspected *B*. *cereus* isolates from this study, we performed whole genome sequencing of 109 samples, which were sub-cultured on Chromagar medium (S2 Table). These samples were selected from both 2017 and 2018 harvest years. Genomes were then *de novo* assembled, which generated a median genome size of ~6.2Mb and GC content of ~35% (S4 Table).

**Fig 1:**
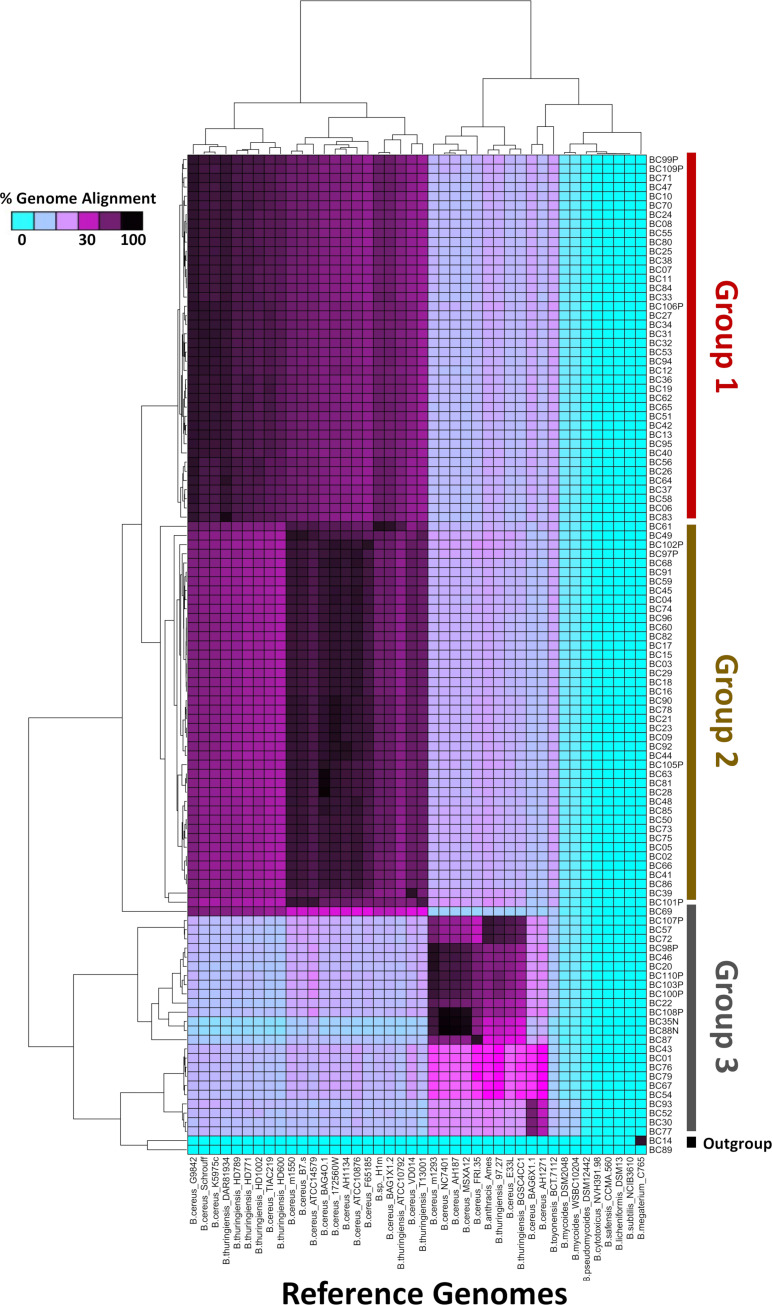
Heatmap of percent genome alignment for *B*. *cereus* suspected samples to available *Bacillus* reference genomes. The vertical axis indicates group assignments and sample identifiers. The horizontal axis indicates representative reference genomes available from NCBI from S7 Table. The heatmap scale reflects percent genome alignment. Hierarchical clustering of the data by ‘hclust’ is represented by dendrograms. The colour of the groups (right vertical axis), and samples within, are the same as in Figs 2 and 3.

**Fig 2:**
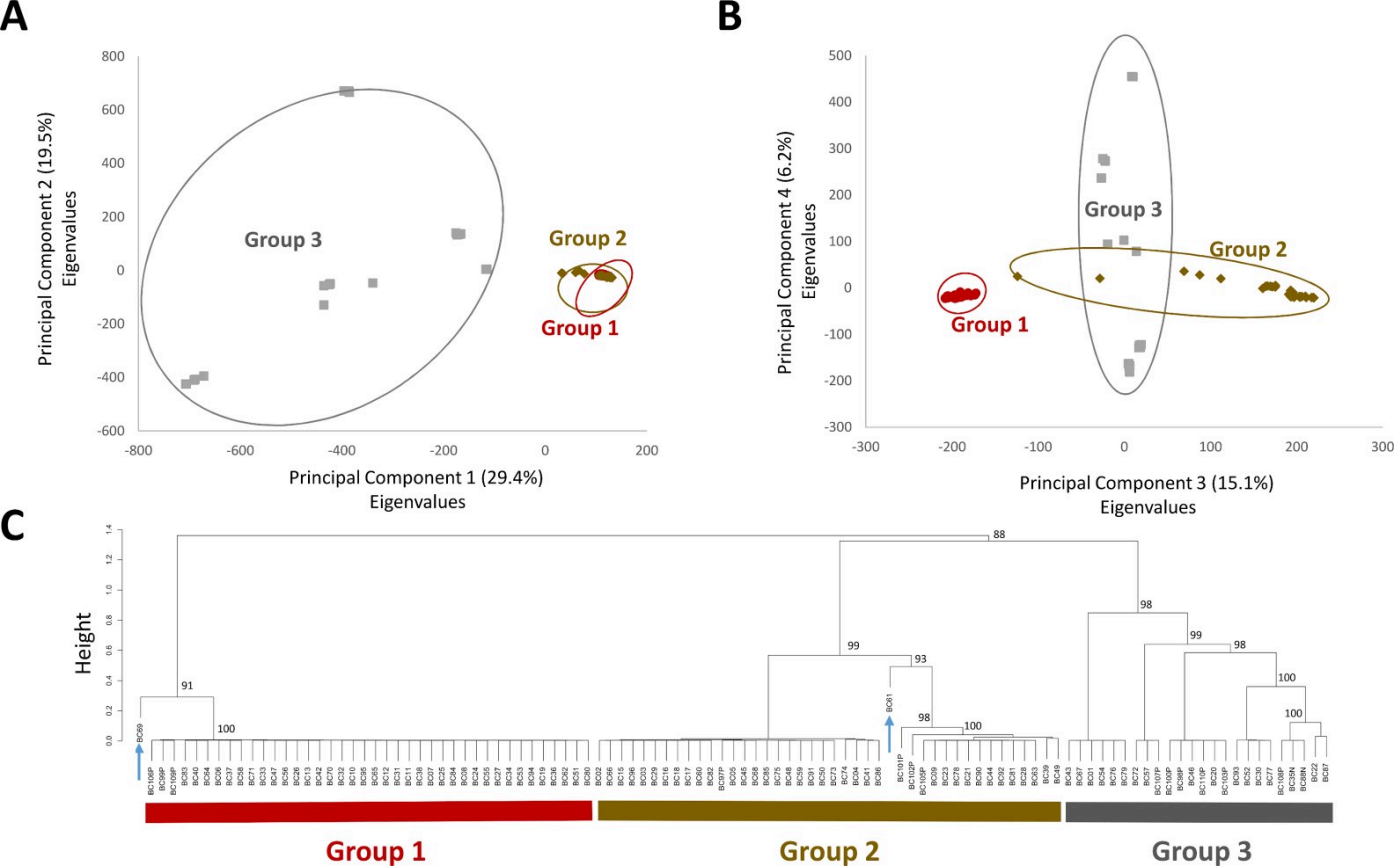
Analysis of principal components for nucleotide variants identified by whole genome alignments. Variants were identified based on whole genome alignments using MUMmer. Sample relationships based on the eigenvalues from the first two principal components (A), and the third and fourth principal components (B) are shown (S8 Table). Percentage of variance explained by each component is indicated in brackets. C, Dendogram of the variant-based principal component analysis using ‘prcomp’ and clustering by ‘pvclust’, bootstrapping values indicate the approximated unbiased percent likelihood based on 1,000 clustering iterations. Blue arrows indicated samples BC61 and BC69, which occupy unique branches. The colour of the groups, and samples within, are the same as in Figs 1 and 3.

To identify candidate species for each sample, genomes were compared to available genomes from the RefSeq database at NCBI using RefSeq_masher and from MLST analysis (S5 and S6 Tables). The majority of the samples were identified to be similar to species within the *B*. *cereus* group by RefSeq_masher and were identified to be from the *B*. *cereus* scheme by MLST (S5 and S6 Tables). To validate the species assignments and better identify the relationship between the samples and those from publically available genomes, we downloaded the genomes for the top RefSeq_masher hits and performed whole genome alignments to each of our newly assembled genomes by MUMmer (Fig 1; S7 Table). Whole genome alignments confirmed that that the majority of the samples had high genome alignment to those from *B*. *cereus* group sequenced from other studies. Most of the samples fell within two closely related groups, containing 40 (Group 1) and 43 (Group 2) samples, respectively. The remainder of the samples (Group 3) were much more diverse and were more distantly related to Groups 1 and 2 (Fig 1). The sample relationships were in agreement with a principal components analysis of nucleotide variants, which demonstrated a close relationship between Groups 1 and 2, with some separation by Principal Component 3 (Fig 2). Group 3 was much more diverse and contained several subgroups (Figs 1 and 2).

Three of the samples (BC35N, BC88N, and BC108P) had over 97% genome alignment to the reference genomes for NC7401 and AH187, which are emetic strains of *B*. *cereus* and were isolated from cases of foodborne illness [43]. Four strains (BC93, BC52, BC30, and BC77) had ~70% genome alignment to *B*. *wiedmannii* strain BAG6X1, and formed a subgroup within Group 3. For some of the other samples, the species assignment was less clear. For example, BC69 aligned well to Group 1 and 2; however, alignment was too low for it to be clearly placed in these species, with the greatest alignment (~65%), being to strains G9842 and Lr7/2 (Fig 2C blue arrow; S7 Table). Curiously, the genome size was quite large at 8.9Mb, but the GC content was 38%. Likewise, six strains (BC87, BC43, BC01, B76, BC 79, and BC67) all had reduced alignment to available references; however, unlike BC69, these strains had poor alignment to most Group 1 and 2 references, but had some alignment to Group 3 references (Figs 1 and 2).

A small set of the samples were identified by RefSeq_masher to be bacteria from other genera or species, including *Enterococcus*, *Actinobacter*, *Brevibaccilus*, and other *Bacillus* species (S5–S7 Tables). The presence of other bacterial species in the samples was further supported by differences in genome size and GC content when compare to those that were identified to be from the *B*. *cereus* group, suggesting that multiple bacteria may be present in some samples (S4 Table). Curiously, two of the samples appear to be pure samples from more distant bacteria, including one isolate related to *Brevibacillus* sp (BC89) and one isolate of *B*. *megaterium* (BC14), which formed an outgroup in our genome alignment analyses (Fig 1) and were excluded from the variant analysis (Fig 2; S5–S7 Tables). Both of these organisms have previously been reported in the soil/rhizosphere and contribute to complex microbe-microbe and/or microbe-plant interactions, with *Brevibacillus* sp. having biocontrol and bioremediation potential and *B*. *megaterium* promoting plant growth [44, 45]. While detection and sequencing of these off-target organisms was both interesting and unexpected, the majority of the samples that were sequenced contained diverse strains of *B*. *cereus* (Figs 1 and 2).

### Analysis of genome and gene content

*B*. *cereus* is genetically diverse and known for its presence/absence variation in genes involved in disease, toxin production, and antimicrobial resistance (AMR) [33–36]. This variation is partly due to the presence of plasmids, which are very common in *B*. *cereus*; some *B*. *thuringiensis* strains may carry more than 15 plasmids [46]. In addition, plasmid size varies greatly and the plasmid profile of the different strains does not always match their phylogeny. The *B*. *cereus* genome assemblies were analysed for plasmids using PlasmidFinder for replicon typing/subtyping (S9 Table). PlasmidFinder supported the presence of plasmids in the genomes, including pBMB67 in BC26, which was previously identified in *B*. *thuringiensis* and was implicated in cell–cell signaling and regulation of cellular processes [47].

**Fig 3:**
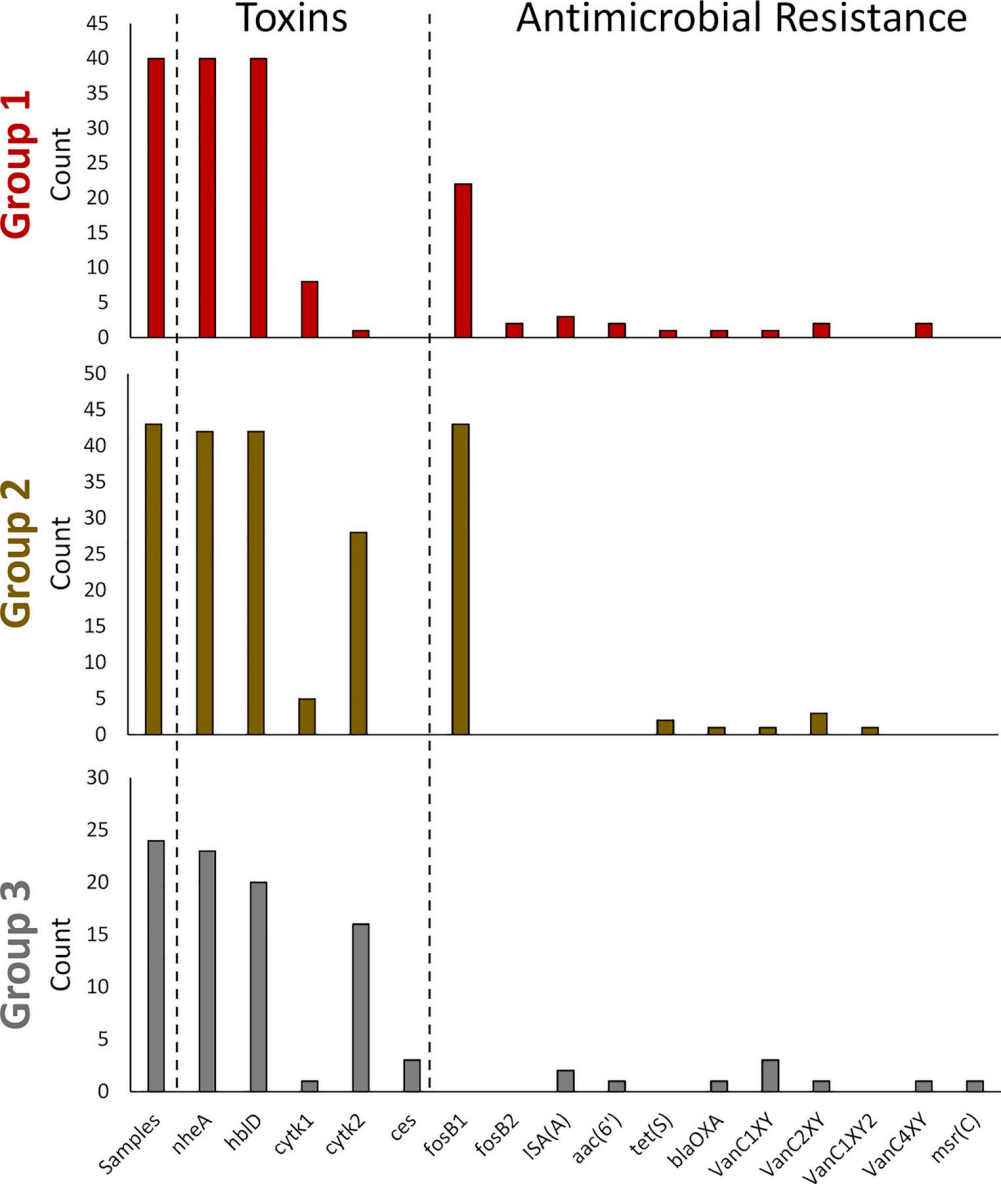
Toxin and AMR genes identified in *B*. *cereus* samples. The total number (count) of samples, toxin genes (real-time PCR) and AMR results are presented for each group. Details on each sample are in S10 Table. The group assignments are indicated by the same colours used in Figs 1 and 2.

Some *B*. *cereus* strains are known to produce enterotoxin and emetic toxins that have implications in disease; as such, we performed real-time PCR, BLAST, and Kma analyses of some toxin genes. *B*. *cereus* toxins that are associated with foodborne illness include hemolysin BL (partially encoded by *hbLD*), the non-hemolytic enterotoxin Nhe (partially encoded by *nheA*), cytotoxin K (*cytk*), and cereulide (*ces*) (S10 Table) [34–36]. The agreement in the PCR and tBLASTn results were 90.8% for *hblD*, 97.2% for *nheA*, 84.4% for *cytk1*, 67.0% for *cytk2*, and 100% for *ces*. This was similar to the analysis comparing the PCR and Kma results, which were in 89.0, 98.2, 87.2 and 67.9 and 100% agreement with the PCR results, respectively. The high concordance in the results for *hblD*, *nheA*, and *ces* demonstrate the reliability of PCR-based methods in the detection of most of these genes (S10 Table). The reduced agreement in the *cytk* results may be because of additional diversity in the *cytk* alleles. Our real-time PCR results showed that a large proportion of the *B*. *cereus* group strains from grain carry the *nheA* (96.33%) and *hbID* (94.5%) genes, while 12.8%, 42.2%, and 2.8% strains carried *cytk1*, *cytk2*, and *ces* respectively (Fig 3). The frequent occurrence of *nheA* and of *hblD* genes was comparable with the previous studies [48, 49]. The *cytK* gene was less frequent than *nhe*, and *hbl*, which is also in agreement with previous studies of food (37%) and diarrheal type of food poisoning (73%) [48, 49]. Curiously, *cytK2* was less common in samples from Group 1, despite their genomic similarity to Group 2 (Fig 3). The *ces* gene, encoding emetic toxin and known as cereulide synthetase, was detected in only three samples of CWRS, one from 2017 and two from 2018 (BC35N, BC88N, and BC108P). These are the three sample samples that clustered together within Group 3 and had high genome alignment to the emetic strains NC7401 and AH187 (Figs 1–3). This finding indicates that emetic toxin producing isolates of *B*. *cereus* on grain are rare.

To investigate coincidence of toxins, all strains were divided into 11 different toxin genes profiles according to the presence or absence of toxin genes (Table 2). Pattern I (51 isolates, 46.36%) was the most common pattern and contains enterotoxin genes (*nheA* and *hblD*), which was followed by pattern II (35 isolates, 32.11%), which carried both *nheA* and *hblD* plus *cytk2* genes. This finding is comparable to other studies that identified that the majority of strains (79 of 147; 53.7%) encoded the *nheAB* and *hblDA* genes. Patterns V, VI, VIII, X and XI were rare and each pattern consisted only one isolate (0.91%). Patterns II, III, IV, V, X and XI were positive for 3 or more enterotoxin genes, including *nheA*, *hblD*, *ces* or *cytK*s. Interestingly, the cereulide positive isolates belonged to three different toxin patterns (Patterns V, VI and X), despite their close relationships.

**Table 2:** Different toxin gene profiles of *B*. *cereus* strains used in this study.

Pattern	*nheA*	*hbID*	*cytK1*	*cytk2*	*ces*	Count (%)
I	+	+	-	-	-	51 (46.36%)
II	+	+	-	+	-	35 (32.11%)
III	+	+	+	-	-	8 (7.27%)
IV	+	+	+	+	-	5 (4.55%)
V	+	+	-	-	+	1 (0.91%)
VI	+	-	-	-	+	1 (0.91%)
VII	-	+	-	-	-	2 (1.82%)
VIII	-	-	-	+	-	1 (0.91%)
IX	+	-	-	+	-	3 (2.73%)
X	+	-	-	+	+	1 (0.91%)
XI	-	+	+	+	-	1 (0.91%)

^a^ The toxin profiles were assigned to each *B*. *cereus* suspected isolates depend on their presence or absence of targeted toxin genes by real-time PCR.

Our analysis of the genes involved in the production of insecticidal crystals (*Cry* genes; S3 Table) indicate that none of the samples contained the genes investigated. These genes are important for the biopesticide activity *of B*. *thuringiensis* in commercially available products registered for use in Canada [50]. The absence of these genes in the samples from this study suggest that the isolates we recovered are not those from commercial products used in agriculture to control insect pests.

In addition to toxin genes, AMR genes are also important because they could impact the fitness of the bacteria and the treatment strategy in cases of disease. The ResFinder tool was used to identify several AMR genes belonging to different classes of antimicrobials. The majority of *B*. *cereus* samples (61.5%, 67/109) carried either *fosB1* or *fosB1*gene, which confers resistance to fosfomycin (Fig 3; S10 Table). Interestingly, all of the Group 2 samples carried fosfomycin resistance genes, while only a subset of Group 1 samples, and none of the Group 3 samples carried the resistance genes (Fig 3). Genes responsible for resistance to vancomycin (*VanC1XY*, *VanC3XY*, *VanC3XY* and *VanC2XY*) were detected in fifteen isolates, which were broadly distributed across the different *B*. *cereus* groups (Fig 3). Further, five isolates were positive for *ISA(A)*, which is responsible for lincomycin, clindamycin, dalfopristin, pristinamycin 11, virginiamycin, and quinupristin. Tetracycline resistance encoded by *tet(S)*, and the *aac(6’)-Iid* gene that confers resistance to gentamycin, were present in only three isolates each. Taken together, while fosfomycin resistance was common, particularly in samples from Group 2.

The plasmids, toxin genes, and AMR genes identified in this study provide important insights into the status of *B*. *cereus* on grain. However, it is important to note that vancomycin and cefsulodin were used as part of our bacterial enrichment method. Therefore, samples may not proportionately represent the *B*. *cereus* that may be occurring on grain. There may also be additional genes beyond those reported here that provide resistance to these antimicrobial compounds. In addition, contamination by other bacteria, including *Enterococcus* and *Acinetobacter* species, may have resulted in misidentified features in some samples (S5–S7 Tables). Nevertheless, our findings indicate that some toxin genes (*nheA* and *hblD*) and AMR (fosfomycin) are common in the *B*. *cereus* samples on grain, while others, such as *ces* are less frequent.

## Conclusions

Our study is amongst only a few studies that have investigated *B*. *cereus* on raw grain. While we observed that these bacteria were abundant on grain in 2017 and 2018, we noticed a reduction in incidence in later stages of the supply chain. Isolation and whole genome sequencing revealed that these bacteria are diverse and belong to several different species. Further genomic characterization revealed that some of these isolates have the potential to produce toxins; however, strains that produce cereulide are rare. Some of these bacteria may also have resistance to fosfomycin. Since *B*. *cereus* is abundant in our natural environments and can occupy different niches, including roles in promoting plant growth and health, it will be important to continue to describe and monitor this group of bacteria and unravel its diversity.

## Supporting information

S1 Fig Scree plot illustrating the variance as a function of number of components for the nucleotide variants identified by whole genome alignments. Seven components were selected for use in hierarchical clustering analyses.
(TIF)

S1 Table Primer sequences used for the detection of *B*. *cereus* and toxin genes. (XLSX)

S2 Table Origin of samples investigated in this study. (XLSX)

S3 Table Genes included in the comparative genomic analysis by tBLASTn and Kma. (XLSX)

S4 Table Assembly statistics. (XLSX)

S5 Table Top five matching genomes predicted by RefSeq_masher matches. (XLSX)

S6 Table MLST analysis of the sequenced genomes. (XLSX)

S7 Table Percent genome alignment to NCBI reference genome sequences. (XLSX)

S8 Table Variant-based principal components. (XLSX)

S9 Table Plasmids and antibiotic resistence genes identified by PlasmidFinder and ResFinder. (XLSX)

S10 Table Samples that tested positive and negative for toxin genes. (XLSX)

S11 Table List of NCBI accession numbers of the bacterial isolates. (XLSX)

## References

[1] Zeng Q, Xie J, Li Y, Gao T, Xu C, Wang Q. Comparative genomic and functional analyses of four sequenced Bacillus cereus genomes reveal conservation of genes relevant to plant-growth-promoting traits. Scientific Reports. 2018;8(1):17009. doi: 10.1038/s41598-018-35300-y 30451927 PMC6242881

[2] Ceuppens S, Boon N, Uyttendaele M. Diversity of Bacillus cereus group strains is reflected in their broad range of pathogenicity and diverse ecological lifestyles. FEMS Microbiology Ecology. 2013;84(3):433–50. doi: 10.1111/1574-6941.12110 23488744

[3] Ehling-Schulz M, Frenzel E, Gohar M. Food-bacteria interplay: pathometabolism of emetic Bacillus cereus. Front Microbiol. 2015;6:704–. doi: 10.3389/fmicb.2015.00704 . 26236290 PMC4500953

[4] Ruan L, Crickmore N, Peng D, Sun M. Are nematodes a missing link in the confounded ecology of the entomopathogen Bacillus thuringiensis? Trends in Microbiology. 2015;23(6):341–6. doi: 10.1016/j.tim.2015.02.011 25818004

[5] Biesta-Peters EG, Dissel S, Reij MW, Zwietering MH, in’t Veld PH. Characterization and Exposure Assessment of Emetic Bacillus cereus and Cereulide Production in Food Products on the Dutch Market. Journal of food protection. 2016;79(2):230–8. Epub 2016/01/29. doi: 10.4315/0362-028X.JFP-15-217 . 26818983

[6] Daczkowska-Kozon EG, Bednarczyk A, Biba M, Repich K. BACTERIA OF BACILLUS CEREUS GROUP IN CEREALS AT RETAIL. Polish Journal of Food and Nutrition Sciences. 2009;59(1):53–9.

[7] Messelhäusser U, Frenzel E, Blöchinger C, Zucker R, Kämpf P, Ehling-Schulz M. Emetic Bacillus cereus Are More Volatile Than Thought: Recent Foodborne Outbreaks and Prevalence Studies in Bavaria (2007–2013). BioMed Research International. 2014;2014:465603. doi: 10.1155/2014/465603 24895578 PMC4033357

[8] Molva C, Sudagidan M, Okuklu B. Extracellular enzyme production and enterotoxigenic gene profiles of Bacillus cereus and Bacillus thuringiensis strains isolated from cheese in Turkey. Food Control. 2009;20(9):829–34. 10.1016/j.foodcont.2008.10.016.

[9] Organji SR, Abulreesh HH, Elbanna K, Osman GEH, Khider M. Occurrence and characterization of toxigenic Bacillus cereus in food and infant feces. Asian Pacific Journal of Tropical Biomedicine. 2015;5(7):515–20. 10.1016/j.apjtb.2015.04.004.

[10] Reyes JE, Bastías JM, Gutiérrez MR, Rodríguez Mde L. Prevalence of Bacillus cereus in dried milk products used by Chilean School Feeding Program. Food microbiology. 2007;24(1):1–6. Epub 2006/09/01. doi: 10.1016/j.fm.2006.04.004 . 16943088

[11] Berghofer LK, Hocking AD, Miskelly D, Jansson E. Microbiology of wheat and flour milling in Australia. International Journal of Food Microbiology. 2003;85(1):137–49. doi: 10.1016/s0168-1605(02)00507-x 12810278

[12] Scallan E, Hoekstra RM, Angulo FJ, Tauxe RV, Widdowson M-A, Roy SL, et al. Foodborne illness acquired in the United States—major pathogens. Emerg Infect Dis. 2011;17(1):7–15. doi: 10.3201/eid1701.p11101 . 21192848 PMC3375761

[13] Glasset B, Herbin S, Granier SA, Cavalié L, Lafeuille E, Guérin C, et al. Bacillus cereus, a serious cause of nosocomial infections: Epidemiologic and genetic survey. PloS one. 2018;13(5):e0194346. Epub 2018/05/24. doi: 10.1371/journal.pone.0194346 ; PubMed Central PMCID: PMC5966241. 29791442 PMC5966241

[14] Liu Y, Lai Q, Göker M, Meier-Kolthoff JP, Wang M, Sun Y, et al. Genomic insights into the taxonomic status of the Bacillus cereus group. Scientific Reports. 2015;5(1):14082. doi: 10.1038/srep14082 26373441 PMC4571650

[15] Bhatnagar R, Batra S. Anthrax toxin. Critical reviews in microbiology. 2001;27(3):167–200. Epub 2001/10/13. doi: 10.1080/20014091096738 . 11596878

[16] Bottone EJ. Bacillus cereus, a volatile human pathogen. Clin Microbiol Rev. 2010;23(2):382–98. doi: 10.1128/CMR.00073-09 . 20375358 PMC2863360

[17] Palma L, Muñoz D, Berry C, Murillo J, Caballero P. Bacillus thuringiensis toxins: an overview of their biocidal activity. Toxins. 2014;6(12):3296–325. Epub 2014/12/17. doi: 10.3390/toxins6123296 ; PubMed Central PMCID: PMC4280536. 25514092 PMC4280536

[18] Jiménez G, Urdiain M, Cifuentes A, López-López A, Blanch AR, Tamames J, et al. Description of Bacillus toyonensis sp. nov., a novel species of the Bacillus cereus group, and pairwise genome comparisons of the species of the group by means of ANI calculations. Systematic and applied microbiology. 2013;36(6):383–91. Epub 2013/06/25. doi: 10.1016/j.syapm.2013.04.008 . 23791203

[19] Guinebretière MH, Auger S, Galleron N, Contzen M, De Sarrau B, De Buyser ML, et al. Bacillus cytotoxicus sp. nov. is a novel thermotolerant species of the Bacillus cereus Group occasionally associated with food poisoning. International journal of systematic and evolutionary microbiology. 2013;63(Pt 1):31–40. Epub 2012/02/14. doi: 10.1099/ijs.0.030627-0 . 22328607

[20] LECHNER S, MAYR R, FRANCIS KP, PRÜß BM, KAPLAN T, WIEßNER-GUNKEL E, et al. Bacillus weihenstephanensis sp. nov. is a new psychrotolerant species of the Bacillus cereus group. International journal of systematic and evolutionary microbiology. 1998;48(4):1373–82. doi: 10.1099/00207713-48-4-1373 9828439

[21] Jung MY, Paek WK, Park IS, Han JR, Sin Y, Paek J, et al. Bacillus gaemokensis sp. nov., isolated from foreshore tidal flat sediment from the Yellow Sea. Journal of microbiology (Seoul, Korea). 2010;48(6):867–71. Epub 2011/01/12. doi: 10.1007/s12275-010-0148-0 . 21221948

[22] Jung MY, Kim JS, Paek WK, Lim J, Lee H, Kim PI, et al. Bacillus manliponensis sp. nov., a new member of the Bacillus cereus group isolated from foreshore tidal flat sediment. Journal of microbiology (Seoul, Korea). 2011;49(6):1027–32. Epub 2011/12/29. doi: 10.1007/s12275-011-1049-6 . 22203569

[23] Liu B, Liu GH, Hu GP, Sengonca C, Lin NQ, Tang JY, et al. Bacillus bingmayongensis sp. nov., isolated from the pit soil of Emperor Qin’s Terra-cotta warriors in China. Antonie van Leeuwenhoek. 2014;105(3):501–10. Epub 2013/12/29. doi: 10.1007/s10482-013-0102-3 . 24370979

[24] Miller RA, Beno SM, Kent DJ, Carroll LM, Martin NH, Boor KJ, et al. Bacillus wiedmannii sp. nov., a psychrotolerant and cytotoxic Bacillus cereus group species isolated from dairy foods and dairy environments. International journal of systematic and evolutionary microbiology. 2016;66(11):4744–53. Epub 2016/08/16. doi: 10.1099/ijsem.0.001421 ; PubMed Central PMCID: PMC5381181. 27520992 PMC5381181

[25] Lotte R, Hérissé A-L, Berrouane Y, Lotte L, Casagrande F, Landraud L, et al. Virulence Analysis of Bacillus cereus Isolated after Death of Preterm Neonates, Nice, France, 2013. Emerging Infectious Disease journal. 2017;23(5):845. doi: 10.3201/eid2305.161788 28418291 PMC5403044

[26] Dierick K, Van Coillie E, Swiecicka I, Meyfroidt G, Devlieger H, Meulemans A, et al. Fatal family outbreak of Bacillus cereus-associated food poisoning. J Clin Microbiol. 2005;43(8):4277–9. doi: 10.1128/JCM.43.8.4277-4279.2005 . 16082000 PMC1233987

[27] Beecher DJ, Schoeni JL, Wong AC. Enterotoxic activity of hemolysin BL from Bacillus cereus. Infect Immun. 1995;63(11):4423–8. doi: 10.1128/iai.63.11.4423-4428.1995 . 7591080 PMC173629

[28] Lund T, Granum PE. Characterisation of a non-haemolytic enterotoxin complex from Bacillus cereus isolated after a foodborne outbreak. FEMS microbiology letters. 1996;141(2–3):151–6. Epub 1996/08/01. doi: 10.1111/j.1574-6968.1996.tb08377.x . 8768516

[29] Lund T, De Buyser M-L, Granum PE. A new cytotoxin from Bacillus cereus that may cause necrotic enteritis. Molecular Microbiology. 2000;38(2):254–61. doi: 10.1046/j.1365-2958.2000.02147.x 11069652

[30] Ehling-Schulz M, Vukov N, Schulz A, Shaheen R, Andersson M, Märtlbauer E, et al. Identification and Partial Characterization of the Nonribosomal Peptide Synthetase Gene Responsible for Cereulide Production in Emetic Bacillus cereus. Applied and Environmental Microbiology. 2005;71(1):105. doi: 10.1128/AEM.71.1.105-113.2005 15640177 PMC544239

[31] Granum PE, Lund T. Bacillus cereus and its food poisoning toxins. FEMS microbiology letters. 1997;157(2):223–8. Epub 1998/01/22. doi: 10.1111/j.1574-6968.1997.tb12776.x . 9435100

[32] Ehling-Schulz M, Messelhäusser U. Bacillus "next generation" diagnostics: moving from detection toward subtyping and risk-related strain profiling. Front Microbiol. 2013;4:32. Epub 2013/02/27. doi: 10.3389/fmicb.2013.00032 ; PubMed Central PMCID: PMC3579190. 23440299 PMC3579190

[33] Chang YH, Shangkuan YH, Lin HC, Liu HW. PCR assay of the groEL gene for detection and differentiation of Bacillus cereus group cells. Appl Environ Microbiol. 2003;69(8):4502–10. Epub 2003/08/07. doi: 10.1128/AEM.69.8.4502-4510.2003 ; PubMed Central PMCID: PMC169126. 12902235 PMC169126

[34] Wehrle E, Didier A, Moravek M, Dietrich R, Märtlbauer E. Detection of Bacillus cereus with enteropathogenic potential by multiplex real-time PCR based on SYBR Green I. Molecular and cellular probes. 2010;24(3):124–30. Epub 2009/12/01. doi: 10.1016/j.mcp.2009.11.004 . 19944752

[35] Fricker M, Messelhäußer U, Busch U, Scherer S, Ehling-Schulz M. Diagnostic Real-Time PCR Assays for the Detection of Emetic Bacillus cereus Strains in Foods and Recent Food-Borne Outbreaks. Applied and Environmental Microbiology. 2007;73(6):1892–8. doi: 10.1128/AEM.02219-06 17259359 PMC1828801

[36] Guinebretiere M-H, Fagerlund A, Granum PE, Nguyen-The C. Rapid discrimination of cytK-1 and cytK-2 genes in Bacillus cereus strains by a novel duplex PCR system. FEMS microbiology letters. 2006;259(1):74–80. doi: 10.1111/j.1574-6968.2006.00247.x 16684105

[37] Stenfors Arnesen LP, Fagerlund A, Granum PE. From soil to gut: Bacillus cereus and its food poisoning toxins. FEMS Microbiology Reviews. 2008;32(4):579–606. doi: 10.1111/j.1574-6976.2008.00112.x 18422617

[38] Bolger AM, Lohse M, Usadel B. Trimmomatic: a flexible trimmer for Illumina sequence data. Bioinformatics. 2014;30(15):2114–20. Epub 04/01. doi: 10.1093/bioinformatics/btu170 . 24695404 PMC4103590

[39] Koren S, Walenz BP, Berlin K, Miller JR, Bergman NH, Phillippy AM. Canu: scalable and accurate long-read assembly via adaptive k-mer weighting and repeat separation. Genome Research. 2017. doi: 10.1101/gr.215087.116 28298431 PMC5411767

[40] Gurevich A, Saveliev V, Vyahhi N, Tesler G. QUAST: quality assessment tool for genome assemblies. Bioinformatics. 2013;29(8):1072–5. Epub 2013/02/21. doi: 10.1093/bioinformatics/btt086 ; PubMed Central PMCID: PMC3624806. 23422339 PMC3624806

[41] Seemann T. Prokka: rapid prokaryotic genome annotation. Bioinformatics. 2014;30(14):2068–9. doi: 10.1093/bioinformatics/btu153 24642063

[42] Marçais G, Delcher AL, Phillippy AM, Coston R, Salzberg SL, Zimin A. MUMmer4: A fast and versatile genome alignment system. PLOS Computational Biology. 2018;14(1):e1005944. doi: 10.1371/journal.pcbi.1005944 29373581 PMC5802927

[43] Carroll LM, Wiedmann M, Mukherjee M, Nicholas DC, Mingle LA, Dumas NB, et al. Characterization of Emetic and Diarrheal Bacillus cereus Strains From a 2016 Foodborne Outbreak Using Whole-Genome Sequencing: Addressing the Microbiological, Epidemiological, and Bioinformatic Challenges. Front Microbiol. 2019;10(144). doi: 10.3389/fmicb.2019.00144 30809204 PMC6379260

[44] Zhou C, Ma Z, Zhu L, Xiao X, Xie Y, Zhu J, et al. Rhizobacterial Strain Bacillus megaterium BOFC15 Induces Cellular Polyamine Changes that Improve Plant Growth and Drought Resistance. International Journal of Molecular Sciences. 2016;17(6):976. doi: 10.3390/ijms17060976 27338359 PMC4926508

[45] Mallick I, Hossain ST, Sinha S, Mukherjee SK. Brevibacillus sp. KUMAs2, a bacterial isolate for possible bioremediation of arsenic in rhizosphere. Ecotoxicology and Environmental Safety. 2014;107:236–44. doi: 10.1016/j.ecoenv.2014.06.007 25011120

[46] Reyes-Ramírez A, Ibarra JE. Plasmid patterns of Bacillus thuringiensis type strains. Applied and environmental microbiology. 2008;74(1):125–9. Epub 11/16. doi: 10.1128/AEM.02133-07 . 18024687 PMC2223206

[47] Chao L, Qiyu B, Fuping S, Ming S, Dafang H, Guiming L, et al. Complete nucleotide sequence of pBMB67, a 67-kb plasmid from Bacillus thuringiensis strain YBT-1520. Plasmid. 2007;57(1):44–54. doi: 10.1016/j.plasmid.2006.06.002 16901541

[48] Guinebretière M-H, Broussolle V, Nguyen-The C. Enterotoxigenic Profiles of Food-Poisoning and Food-Borne Bacillus cereus Strains. J Clin Microbiol. 2002;40(8):3053–6. doi: 10.1128/JCM.40.8.3053-3056.2002 12149378 PMC120679

[49] Kindle P, Etter D, Stephan R, Johler S. Population structure and toxin gene profiles of Bacillus cereus sensu lato isolated from flour products. FEMS microbiology letters. 2019;366(20). doi: 10.1093/femsle/fnz240 31769798

[50] Bonis M, Felten A, Pairaud S, Dijoux A, Maladen V, Mallet L, et al. Comparative phenotypic, genotypic and genomic analyses of Bacillus thuringiensis associated with foodborne outbreaks in France. PloS one. 2021;16(2):e0246885–e. doi: 10.1371/journal.pone.0246885 . 33607651 PMC7895547

