# Supplementary figures and images for: Profiling of Bacillus cereus on Canadian grain
Source: PLoS One. 2021 Nov 4;16(11):e0259209. doi: 10.1371/journal.pone.0259209 (PMC8568128; doi:10.1371/journal.pone.0259209)

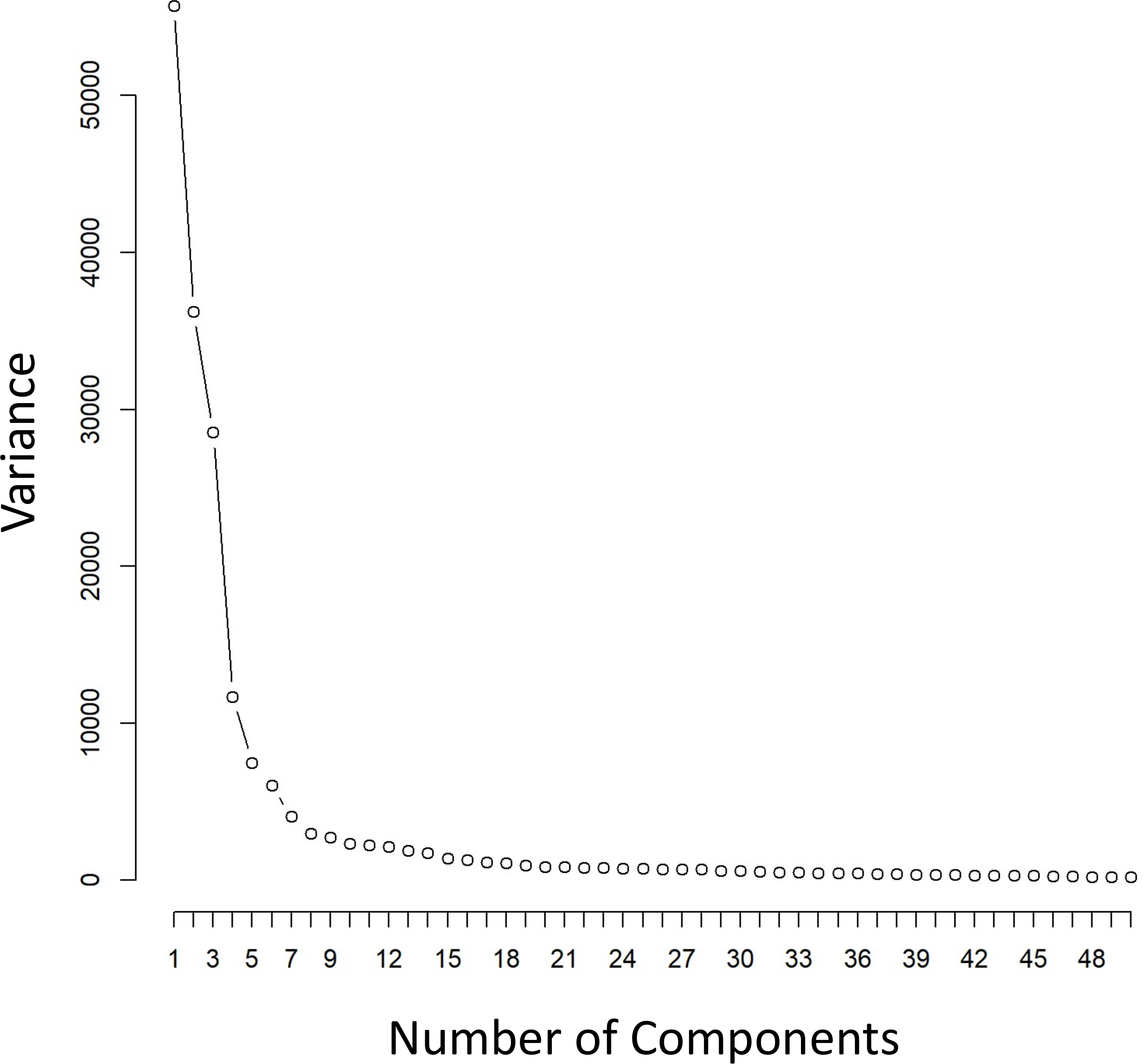

Supplement: S1 Fig — Seven components were selected for use in hierarchical clustering analyses. (TIF) [file pone.0259209.s001.tif]
